# The roles of microglia and astrocytes in inflammasomes and neurological disorders

**DOI:** 10.4103/NRR.NRR-D-24-01574

**Published:** 2025-08-13

**Authors:** Yuze Xia, Yimin Huang, Yuan Liu, Xincheng Zhang, Huayu Kang, Yanchao Liu, Chenxuan Yu, Chao Gan, Huaqiu Zhang

**Affiliations:** 1 Department of Neurosurgery, Tongji Hospital of Tongji Medical College of Huazhong University of Science and Technology, Wuhan, Hubei Province, China; 2 Hubei Key Laboratory of Neural Injury and Functional Reconstruction, Huazhong University of Science and Technology, Wuhan, Hubei Province, China

**Keywords:** astrocytes, cerebrovascular diseases, inflammasomes, inflammation, microglia, neurodegenerative diseases, neuroinflammation, neurological disorders

## Abstract

Inflammasomes, a category of protein complexes, recognize exogenous pathogens and endogenous tissue damage. In response, they induce inflammatory responses and pyroptosis, and are involved in both innate immunity and the regulation of adaptive immunity, with significant effects in disease and health. Neuroinflammation is closely related to neurological disorders. Nervous system homeostasis is primarily regulated by glial cells, with microglia and astrocytes playing a dual role in both neuroprotection and neurotoxicity. Recent studies highlight the importance of microglia and astrocytes within the central nervous system in mediating neuroinflammation associated with neuropsychiatric diseases. In particular, the role of inflammasomes in glial cells and neuroinflammation has garnered growing attention. This review classifies inflammasomes and their activation mechanisms as well as explores their involvement in the activation of microglia and astrocytes in various neurological diseases, aiming to contribute a deeper understanding of the pathogenesis of neurodegenerative disease and brain injury and identification of novel therapeutic targets.

## Introduction

Innate immunity. which constitutes the initial defense response against infection and is evolutionarily conserved in mammals, relies on physical, chemical, and biological barriers, as well as immune cells and effector molecules (Shokal and Eleftherianos, 2017; Li and Wu, 2021). Unlike adaptive immunity, innate immune responses are prompt but nonspecific (Heneka et al., 2018). Effector cells of the innate immune system recognize pathogen-associated molecular patterns (PAMPs) and damage-associated molecular patterns (DAMPs) through pattern-recognition receptors. Subsequently, they execute immune functions, including complement activation, pro-inflammatory processes, and phagocytosis. PAMPs are highly conserved exogenous components expressed only in microorganisms, and include lipopolysaccharide (LPS), mannose, and lipoteichoic acid (Potrykus et al., 2021). DAMPs are molecules, e.g., heat shock proteins, biglycan, and S100 proteins, that are released by damaged tissues in response to insults such as inflammation, necrosis, and stress (Roh and Sohn, 2018). Pattern-recognition receptors are a class of germline-encoded receptors that exert immune effects, such as anti-tumor effects and immune responses against infection, by recognizing PAMPs and DAMPs (Takeuchi and Akira, 2010; Brubaker et al., 2015; Man and Jenkins, 2022). Pattern-recognition receptors can be classified into five main clusters based on protein structural domain homology, namely, absent in melanoma-2 (AIM-2)-like receptors, C-type lectin receptors, nucleotide oligomerization domain-like receptors (NLRs), retinoic acid-inducible gene-I-like receptors, and Toll-like receptors (TLRs) (Takeuchi and Akira, 2010). Inflammasomes are important components of the innate immune system and are also responsible for regulating adaptive immunity (Deets and Vance, 2021). AIM-2 and NLRs participate in the formation of inflammasomes.

Glial cells (GCs) are vital parts of the nervous system that play important roles in neuronal survival, synapse formation and neurotransmitter metabolism (Jessen, 2004). Astrocytes, oligodendrocytes, microglia, and ependymal cells are involved in the central nervous system (CNS), while Schwann cells and satellite cells play roles in the peripheral nervous system. GCs—the most widespread and numerous cells in the CNS—establish extensive and intricate connections with other cells (Yang and Zhou, 2019). Under homeostasis, GCs play important physiological roles in ensuring the structural and functional integrity of the nervous system. In general, microglia, a type of GC present in the CNS, can promote neural stem cell growth (Matsui and Mori, 2018) and myelin development (McNamara et al., 2023), resist infection (Borst et al., 2021), and eliminate dysfunctional or senescent neurons (Nayak et al., 2014; Fricker et al., 2018). Besides, astrocytes—another type of GC of the CNS—are thought to be a potential target in the treatment of neurological disorders owing to their involvement in synaptic messaging, connections with other nerve cells, role in forming the blood–brain barrier (BBB), and neuron-protective functions (Liu and Chopp, 2016; Zhou et al., 2019). However, under abnormal pathological conditions, GCs can contribute to the progression of diseases including glioma (Radin and Tsirka, 2020), Alzheimer’s disease (AD) (Hansen et al., 2018), and ischemic stroke (IS) (Xu et al., 2020).

Microglia and astrocytes play crucial roles in the neuroinflammatory response observed in neurodegenerative diseases and brain injury (Karve et al., 2016; Kwon and Koh, 2020; Liu et al., 2020a). Furthermore, inflammasome activation in the CNS is involved in neuroinflammation (Heneka et al., 2018; Rutsch et al., 2020). In this review, we explore the interaction between inflammasomes and two essential GCs in the CNS: microglia and astrocytes. Our focus lies on elucidating the mechanisms by which inflammasomes play a role in neuroinflammation, describing the activation of inflammasomes by microglia and astrocytes, and summarizing their mutual influence in neurological diseases, with the aim of providing novel perspectives and insights for the clinical treatment of neurodegenerative disease and brain injury.

## Literature Search Strategy

For this narrative review, studies published from 1984 to 2025, mainly from 2020 to 2025, were included. Literature searches were performed on PubMed using the following keywords: inflammasome, microglia, astrocyte, Alzheimer’s disease, Parkinson’s disease, ischemic stroke, cerebral venous sinus thrombosis, subarachnoid hemorrhage, intracerebral hemorrhage, migraine, depression, experimental autoimmune encephalomyelitis, amyotrophic lateral sclerosis. The articles included in this review were selected based on their relevance to the topic. After removing irrelevant and duplicate studies from the retrieved studies, the title and abstract of each article were read in a preliminary screening, and articles that were not highly relevant or were deemed out of scope were deleted.

## Inflammasomes

**Figure 1:**
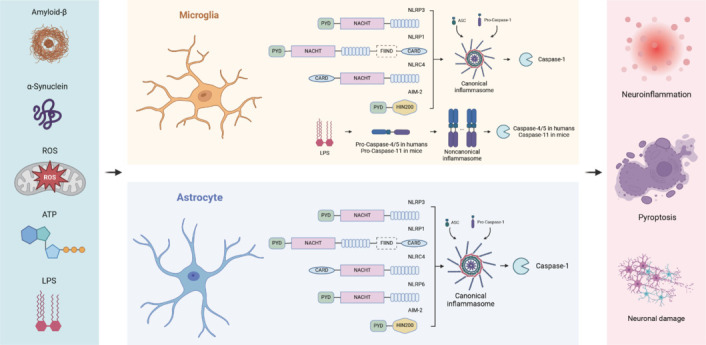
Comparison of inflammasome activation in microglia and astrocytes. The canonical inflammasomes comprise NLRP3, NLRP1, NLRC4, NLRP6, and AIM-2. NACHT and leucine-rich repeats are common features of NLRs. In contrast, NLRP3 and NLRP6 feature a PYD motif, while NLRC4 has a CARD motif at the N-terminal. Additionally, NLRP1 possesses a FIIND and a CARD motif in its C-terminal. AIM-2 consists of both a PYD motif and a HIN200 protein domain. Damage-associated molecular patterns and pathogen-associated molecular patterns such as amyloid-β, α-synuclein, and LPS can activate inflammasomes, leading to neuroinflammation, pyroptosis, and neuron damage. In microglia, NLRP3, NLRP1, NLRC4, AIM-2, and noncanonical inflammasomes are activated, with noncanonical inflammasomes being activated by LPS stimulation. In astrocytes, NLRP3, NLRP1, NLRC4, NLRP6, and AIM-2 are activated. Canonical inflammasomes activate caspase-1, while noncanonical inflammasomes activate caspase-4/5 in humans and caspase-11 in mice. Created with BioRender.com (https://BioRender.com/wjsdtji). ASC: Apoptosis-associated speck-like protein; CARD: caspase recruitment domain; FIIND: function-to-find domain; HIN200: 200-amino-acid repeat; LPS: lipopolysaccharide; NACHT: nucleotide-binding and oligomerization domain; NLRs: nucleotide oligomerization domain-like receptors; PYD: pyrin domain; ROS: reactive oxygen species.

Inflammasomes, initially identified in 2002 (Martinon et al., 2002), are a family of protein complexes that function as intrinsic intracellular immunoreceptors for PAMPs and DAMPs (Noonin and Thongboonkerd, 2021; Seok et al., 2021). Subsequent to the recognition of PAMPs or DAMPs, recruitment and activation of caspase-1 and caspase-11 occur, which induces the maturation and secretion of interleukin (IL)-1β and IL-18, leading to pro-inflammation and pyroptosis (an inflammatory program of cell death) (Lamkanfi and Dixit, 2014; Burdette et al., 2021). Caspases, a group of endoproteases, play a role in modulating apoptosis and inflammation. Inflammasomes can be categorized into two types based on the activated caspases: canonical inflammasomes (involving caspase-1) and noncanonical inflammasomes (involving caspase-4/5 in humans or caspase-11 in mice) (Lamkanfi and Dixit, 2014; Downs et al., 2020; Li et al., 2021b; **Figure 1**).

## Canonical Inflammasome

Each canonical inflammasome is named after the protein scaffold of AIM-2-like receptors or NLRs (Lamkanfi and Dixit, 2012). Although the structure and function of some of these inflammasomes remain poorly understood, NLRP3, NLRP1, NLRC4, NLRP6, and AIM-2 have undergone more thorough study. We will elaborate on these inflammasomes in the following sections.

### NLRP3

NLRP3—the most comprehensively investigated inflammasome to date—consists of three parts: the receptor protein NLRP3, the adaptor protein apoptosis-associated speck-like protein (ASC), and the effector protein procaspase-1. The receptor protein NLRP3 is composed of three key domains: C-terminal leucine-rich repeats for sensing stimulation and intermediate nucleotide-binding; the oligomerization domain (NACHT)—the sole protein structural domain shared by all NLRs—enabling ATP-dependent self-oligomerization; and the N-terminal pyrin domain (PYD) that interacts with ASC, facilitating protein-protein interactions (Schroder and Tschopp, 2010; He et al., 2016a; Huang et al., 2021b). ASC is made up of a PYD, which homotypically interacts with the PYD of NLRP3 receptor, and a caspase recruitment domain (CARD), which recruits caspase-1 when activated (Lamkanfi and Dixit, 2014). NEK7 is a key regulator of NLRP3 inflammasome activation (He et al., 2016b; Shi et al., 2016; Sharif et al., 2019; Sharma and Kanneganti, 2021). NLRP3 can recognize multiple PAMPs (such as LPS, viral RNAs, and toxins) (Dowling and O’Neill, 2012; Huang et al., 2021b) or DAMPs (such as amyloid-β [Aβ] (Halle et al., 2008), hyaluronan (Yamasaki et al., 2009), and ATP (Ratajczak et al., 2019)). The recognition of these PAMPs and DAMPs is followed by the priming and assembly of the NLRP3 inflammasome (Zhao et al., 2021). NLRP3 activation depends on three primary components: ions, mitochondria, and lysosomes. First, K^+^ outflows through the P2X7 channel pore, K^+^-H^+^-ionophore, or incomplete cell membrane (Gong et al., 2018). The decreased cytoplasmic concentration of K^+^ is related to the activation of NLRP3 and caspase-1 (Munoz-Planillo et al., 2013). Cl^−^ efflux through the chloride intracellular channel, concurrent with the decrease of intracellular K^+^ concentration, induces NLRP3 activation as well (Gong et al., 2018; Koumangoye, 2022). Similarly, Ca^2+^ plays a role in activating NLRP3. Calcium-sensing receptor signaling initiates Ca^2+^ release from the endoplasmic reticulum through the inositol trisphosphate pathway; this results in elevated concentrations of cytosolic Ca^2+^ and induces NLRP3 activation (Lee et al., 2012). Notably, although a decrease in extracellular Na+ concentration may inhibit NLRP3 activation to some extent, and Na^+^ influx can depolarize cell membranes, Na^+^ is not necessary for NLRP3 activation (Munoz-Planillo et al., 2013). In addition to ion flow, mitochondria are an important contributor to NLRP3 activation. Mitochondria regulate the production of reactive oxygen species (ROS) through voltage-dependent anion channels, which in turn activates NLRP3. Meanwhile, the inhibition of mitophagy increases the concentration of mitochondrial ROS (Zhou et al., 2011). The flap-structure-specific endonuclease 1 fragments oxidized mitochondrial DNA and these small fragments leave the mitochondria via mitochondrial permeability transition pores and voltage-dependent anion channels-dependent channels, leading to activation of cytoplasmic NLRP3 (Xian et al., 2022). Interestingly, mitochondria-associated membrane and mitochondrial proteins will assist NLRP3 assembly and activation (Swanson et al., 2019). Finally, the release of cathepsins following lysosomal membrane permeabilization promotes NLRP3 activation (Serrano-Puebla and Boya, 2016). Upon activation of NLRP3, procaspase-1 is recruited to self-cleave to form caspase-1, triggering IL-1β and IL-18 maturation and activation (Huang et al., 2021b).

### NLRP1

NLRP1 is ubiquitous in most mammalian species. Humans possess only one *NLRP1* gene which can be alternative-spliced to produce seven isoforms (Yu et al., 2018); in contrast, mice have three NLRP1-encoding genes: *Nlrp1a*, *Nlrp1b*, and *Nlrp1c* (Lamkanfi and Dixit, 2014; Chavarria-Smith and Vance, 2015). In humans, from N-terminal to C-terminal, NLRP1 comprises PYD, NACHT, leucine-rich repeats, central function-to-find domain which performs autolytic hydrolysis that is crucial to NLRP1 activation (Finger et al., 2012), and CARD. In mice, apart from the absence of PYD, the structure of NLRP1b is similar to that of human NLRP1 (Schroder and Tschopp, 2010; Chavarria-Smith and Vance, 2015). Following autoproteolysis of the function-to-find domain, NLRP1 generates two polypeptide chains: C-terminal NLRP1 and N-terminal NLRP1. The C-terminal NLRP1 chain possesses inflammasome assembly activity and can associate with N-terminal NLRP1 or the uncut full-length NLRP1 to produce an auto-inhibitor, thus regulating its own activity (Yang et al., 2022). Interestingly, some studies suggest that activation of caspase-1 by NLRP1 may not critically require ASC (Faustin et al., 2007; Zhai et al., 2017); however, another study indicates that NLRP1b requires ASC for caspase-1 autoprocessing (Van Opdenbosch et al., 2014). Cryo-electron microscopy reveals that DPP9 acts on C-terminal NLRP1 to inhibit NLRP1 activation; this is dependent on the full-length NLRP1 (Hollingsworth et al., 2021). In addition, the protease activity of DPP9 is a key factor responsible for inhibiting the activation of NLRP1 (Huang et al., 2021a). The NLRP1 inflammasome pathway is associated with cellular communication between neurons and glial cells, and plays a vital role in multiple CNS disorders (Mi et al., 2022; Zhang et al., 2024b). The activation, inhibitory mechanisms, and regulatory effects of NLRP1 require further exploration.

### NLRC4

NLRC4, also known as IPAF, CLAN, and CARD12 (Abdelaziz et al., 2010), is composed of leucine-rich repeats, central NACHT, and CARD, from the C-terminal to the N-terminal (Dowling and O’Neill, 2012). The structure of NLRC4 suggests that its activation may not require the involvement of ASC (Lamkanfi and Dixit, 2014); nevertheless, ASC can enhance the activating effect of NLRC4 (Duncan and Canna, 2018). NLRC4 inflammasomes are regulated by several factors including PKC-δ, DDX17, and HSC70. NLRC4 is involved in the regulation of neuroinflammation in a variety of neurological diseases, contributing to poor prognosis (Zhang et al., 2025). Further research is needed to explore other regulatory mechanisms involving the NLRC4 inflammasome pathway as well as potential therapeutic targets.

### NLRP6

NLRP6—another member of the NLRs family, formerly known as PYPAF5 (Venuprasad and Theiss, 2021)—has a similar structure to NLRP3 (Strowig et al., 2012). NLRP6 inflammasomes, which are expressed in the intestine, liver, lung, kidney, joints, and brain (Venuprasad and Theiss, 2021; Li et al., 2022b), play a significant role in the host immune response. Activation of NLRP6 inflammasomes enhances neuroinflammation and pyroptosis (Huang et al., 2024b; Oladapo et al., 2025; Singh et al., 2025). Interestingly, NLRP6 deficiency may suppress the activation of other inflammasomes such as NLRP3 and NLRC4, thereby reducing neuroinflammation and mitigating CNS damage (He et al., 2024). The mechanisms that regulate NLRP6 activation are currently unclear, and further exploration is required to elucidate their roles and potential in the pathogenesis and treatment of CNS inflammatory diseases.

### Absent in melanoma-2

Unlike inflammasomes assembled by NLRs, receptor protein AIM-2 is composed of hematopoietic interferon-inducible nuclear proteins. AIM-2 features a 200-amino-acid repeat protein domain at the C-terminal capable of binding DNA, and a PYD at the N-terminal (Burckstummer et al., 2009; Lamkanfi and Dixit, 2014; Costa Franco et al., 2019). As with NLRP3, AIM-2 requires the involvement of ASC adaptor protein for activation (Lu et al., 2014). Remarkably, unlike NLRs, which possess the NACHT domain, AIM-2 lacks an oligomerization domain, and the double-stranded DNA serves as a foothold for AIM-2 oligomerization. In its inactive state, AIM-2 may be in a self-inhibited state. Once the double-stranded DNA is identified, the 200-amino-acid repeat domain binds to the double-stranded DNA, which reverses its inhibition and leads to AIM-2 activation (Jin et al., 2012). AIM-2 also plays an important role in CNS disorders, influencing cognition and neural homeostasis (Poh et al., 2022; Chiarini et al., 2024; Yang et al., 2024).

## Noncanonical Inflammasomes

Different from canonical inflammasomes that activate caspase-1, noncanonical inflammasomes mediate caspase-4 and caspase-5 activation in humans and caspase-11 activation in mice (Lamkanfi and Dixit, 2014; Downs et al., 2020; Li et al., 2021b). Specifically, it should be noted that noncanonical inflammasome caspases serve not only as effectors, but also as receptor proteins (Downs et al., 2020; Li et al., 2021b). Although caspase-4/5 and caspase-11 are highly homologous (about 55%), with caspase-4 and caspase-11 being more similar (Downs et al., 2020), they exhibit slight differences in function. For instance, caspase-4 can recognize tetraacylated lipid A, whereas caspase-11 cannot (Lagrange et al., 2018). Surprisingly, IL-18 is a substrate for caspase-4 rather than caspase-11, potentially resulting in different pathways for activated noncanonical inflammasomes between humans and mice (Wright et al., 2022).

Gasdermin D (GSDMD), a well-established and primary effector molecule for caspase-4/5/11 (Wright et al., 2022), undergoes cleavage upon caspase activation to mediate pyroptosis (Shi et al., 2015). Upon cleavage, the produced N-terminal of GSDMD forms large oligomerized membrane pores (Liu et al., 2016; Kovacs and Miao, 2017). Consequently, the normal balance of Na^+^ and K^+^ concentration gradients inside and outside the cell is disrupted. When the cell’s compensatory capacity is exceeded, the cell swells and ruptures, leading to pyroptotic cell death (Shi et al., 2015; Kovacs and Miao, 2017). Bacteria are captured in intracellular traps induced by pyroptosis-triggered pores and subsequently cleared by neutrophils or macrophages (Sauer et al., 2011; Jorgensen et al., 2016; Kovacs and Miao, 2017). Additionally, IL-1β and IL-18 are released from GSDMD pores to induce inflammatory reactions (Shi et al., 2015; Kovacs and Miao, 2017; Iwaniuk and Jablonska, 2023).

The noncanonical inflammasomes in the brain are engaged in the regulation of neuroinflammation (Yi, 2021; Brahadeeswaran et al., 2022; Moonen et al., 2023). Although noncanonical inflammasomes have been studied less extensively than canonical inflammasomes, their significance in inflammation and cell death cannot be overlooked. Further research is necessary to elucidate their activation and signaling.

## Inflammasomes in Microglia and Astrocytes

**Figure 2:**
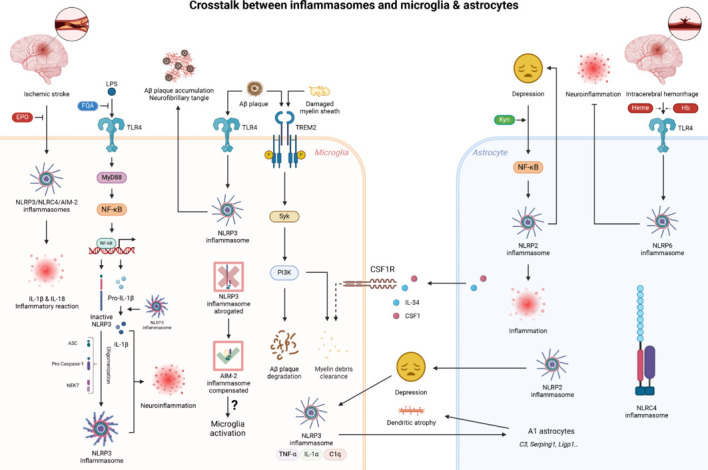
Relationships between microglia, astrocytes, and inflammasomes in ischemic stroke, Alzheimer’s disease, depression, and intracerebral hemorrhage. In microglia, lipopolysaccharide induces the activation of the TLR4/MyD88/NF-κB signaling pathway, leading to the assembly of pro-IL-1β and inactive NLRP3 inflammasomes. Inactive NLRP3, along with ASC, pro-caspase-1, and NEK7, assembles into an active NLRP3 inflammasome, facilitating the conversion of pro-IL-1β to IL-1β and inducing neuroinflammation. Fluoroquinolone antibiotics, such as ciprofloxacin and levofloxacin, inhibit LPS-mediated NLRP3 inflammasome activation. Aβ plaques in Alzheimer’s disease, on one hand, activate TREM2, leading to plaque degradation; on the other hand, they activate TLR4, causing NLRP3 activation, resulting in plaque accumulation and neurofibrillary tangles, thereby exacerbating the disease. Ischemic stroke induces the activation of NLRP3/NLRC4/AIM-2 inflammasomes, triggering the activation of IL-1β and IL-18, resulting in an inflammatory response. Erythropoietin can effectively block inflammasome activation induced by ischemic stroke. Inhibition of the NLRP3 inflammasome in microglia may enhance the expression of the AIM-2 inflammasome; however, the impact of this phenomenon on microglia requires further investigation. In astrocytes, heme and hemoglobin present in hematoma resulting from intracerebral hemorrhage can activate TLR4, causing NLRP6 inflammasome activation and neuroinflammation suppression. Elevated kynurenine levels in depression induce the activation of the NLRP2 inflammasome in astrocytes, which aggravates depressive symptoms and elicits an inflammatory response. NLRP3 activation in microglia induces the formation of neurotoxic astrocytes, leading to dendritic atrophy in depression. Created with BioRender.com (https://BioRender.com/a164k70). Aβ: Amyloid-β; CSF1: colony-stimulating factor 1; CSF1R: colony-stimulating factor 1 receptor; FQA: fluoroquinolone antibiotics; Hb: hemoglobin; Kyn: kynurenine; IL-1β: interleukin-1β; LPS: lipopolysaccharide; NF-κB: nuclear factor kappa B; Syk: spleen tyrosine kinase; TLR4: Toll-like receptor 4; TNF-α: tumor necrosis factor-α; TREM2: the triggering receptor expressed on myeloid cells 2.

Neuroinflammation is a crucial pathological and pathophysiological process in various neuropsychiatric diseases, such as Alzheimer’s disease (AD) (Calsolaro and Edison, 2016), Parkinson’s disease (PD) (Arena et al., 2022), depression (Troubat et al., 2020), and epilepsy (Soltani Khaboushan et al., 2022). Cytokines, including IL-1β, IL-6, IL-23, granulocyte macrophage colony-stimulating factor, tumor necrosis factor, and interferon-γ, are involved in inflammatory processes (Becher et al., 2017). To some extent, acute neuroinflammation is beneficial, while chronic neuroinflammation is detrimental to injured brain tissue (Soltani Khaboushan et al., 2022). Moreover, microglia, astrocytes, and macrophages, along with their interaction with endothelial cells or neurons, play a pivotal role in neuroinflammation (Lyman et al., 2014). Inflammasomes are activated during neuroinflammation in a variety of neurological disorders (Voet et al., 2019). In this section, we introduce the role of microglia, astrocytes and inflammasomes in CNS health and disease (**Figure 2**).

### Microglia and inflammasomes

#### Overview of microglia and neuroinflammation

Although the origin of microglia is controversial, it is generally accepted that these cells originate from the yolk sac during embryonic development. Colony-stimulating factor 1 (CSF1) and IL-34 regulate the proliferation and differentiation of microglia by binding to the CSF1 receptor, leading to a cascade of signaling pathways (Lin et al., 2019). In demyelinating diseases, astrocytes bind to the CSF1 receptor of microglia by expressing CSF1 and IL34, mediating their interaction (Schroder et al., 2023). It is well known that microglia possess macrophage-like innate immune phagocytic function and exhibit immune memory-like function (Wendeln et al., 2018). Simultaneously, microglia communicate with other neurons in the brain, influencing the growth, development, and maturation of neurons (Prinz et al., 2019). However, microglia also express many of the CNS disease-risk genes (Prinz et al., 2019), and under pathological conditions, contribute to the progression of diseases (Zhang et al., 2005; Maphis et al., 2015; Hambardzumyan et al., 2016).

Microglia execute important physiological and pathological functions, relying on a variety of surface proteins. For instance, P2RY12, a G protein-coupled receptor, regulates the migration of microglia in neuroinflammation and the vascular system (Bisht et al., 2021; Gomez Morillas et al., 2021). CX3C chemokine receptor 1, belonging to the chemokine receptor superfamily, is a specific receptor for fractalkine, which participates in synaptic pruning in microglia (Wang et al., 2023a). It is known that microglia exhibit two classical phenotypes, namely, the pro-inflammatory M1 type and the anti-inflammatory M2 type, distinguishable by distinct markers and metabolic states (Orihuela et al., 2016; Yang et al., 2017). Reducing the prevalence of M1 microglia, augmenting the M2 subtype, and facilitating the conversion of M1 to M2 can mitigate neuroinflammation in the CNS, offering a potential avenue for treating neurodegenerative diseases (Yang et al., 2017). Remarkably, low-intensity pulsed ultrasound has the potential to shift microglia from the M1 to M2 phenotype, consequently dampening the inflammatory response (Hsu et al., 2023). Another G protein-coupled receptor, HCAR2—a hydroxycarboxylic acid receptor—exhibits increased expression in microglia following LPS stimulation and can induce the transformation of microglia into the M2 phenotype. Furthermore, HCAR2 may interact with CX3C chemokine receptor 1, leading to the downregulation of pro-inflammatory mRNA induced by fractalkine (Perrone et al., 2023).

#### Microglia-associated inflammasomes in diseases

As previously stated, microglia play a crucial role in neuroinflammation. The triggering receptor expressed on myeloid cells 2 (TREM2) is an innate immune receptor (Schmid et al., 2002) that mediates various functions of microglia. These functions include propelling disease-associated microglia to engulf Aβ plague in AD (Wang et al., 2022a), facilitating the recruitment of glioma-associated microglia and promoting glioma growth (Chen et al., 2023), and enhancing the clearance of myelin debris by microglia and myelin regeneration in multiple sclerosis (Cignarella et al., 2020). Besides, TREM2 regulates the inflammatory reaction in microglia (Kobayashi et al., 2016; Liu et al., 2020b). Nevertheless, microglia can additionally contribute to inflammation through the activation of inflammasomes.

Microglia express TLRs and recognize PAMPs and DAMPs to mediate neuroinflammation (Glass et al., 2010; Stephenson et al., 2018). Similar to noncanonical inflammasomes, TLR4 in microglia is capable of sensing LPS and can be blocked to suppress neuroinflammation (Li et al., 2023a). Fluoroquinolone antibiotics, specifically ciprofloxacin and levofloxacin, can inhibit the binding of LPS to the TLR4-myeloid differentiation protein-2 complex. This inhibition, in turn, reduces the release of pro-neuroinflammatory factors and the activation of nuclear factor kappa B (NF-κB) (Zusso et al., 2019). Therefore, the design or application of anti-neuroinflammatory drugs targeting microglial TLR4 with increased potency, reduced toxicity, and the ability to cross the BBB could be explored to alleviate the symptoms of CNS disorders. NLRP3 is the most generally researched inflammasome in CNS, and is primarily expressed in the microglia of the brain. Upon recognition of PAMPs, such as pathogens, or DAMPs such as damaged cell fragments, microglia produce pro-IL-1β, and the NLRP3 protein is primed through the myeloid differentiation primary response protein MyD88-NF-κB pathway. Subsequently, caspase-1 is activated and converts pro-IL-1β to IL-1β, leading to the secretion of pro-inflammatory factors by microglia, thereby inducing an inflammatory response (Heneka et al., 2018). Additionally, other canonical inflammasomes are expressed in microglia and mediate neuroinflammatory reactions (Freeman et al., 2017; Heinisch et al., 2022).

The association between inflammasomes and microglia is implicated in various CNS disorders. AD is a severely burdensome neurodegenerative disorder characterized by pathological features such as Aβ deposition, neurofibrillary tangles, and neuroinflammation (De Strooper and Karran, 2016; Scheltens et al., 2021). Therapeutic drugs targeting these pathways have shown limited efficacy. Aβ activates NLRP3 in microglia by triggering TLR4 (Liu et al., 2020c). Additionally, NLRP3 activation results in the aggregation of tau protein, potentially leading to an intensified inflammatory response and subsequent degeneration of brain function in patients with AD (Ising et al., 2019). A previous studies has shown that the triggering receptor expressed on myeloid cells-like 2 (TREML2) in microglia is upregulated in response to Aβ stimulation in AD (Sierksma et al., 2020). TREML2 may regulate microglial phenotype transformation and neuroinflammation by promoting the release of inflammatory factors and the NLRP3 inflammasome (Wang et al., 2023b). Thus, TREML2 represents a potential therapeutic target for AD. Spermidine, a polyamine first extracted from semen, is widely distributed in various organs of the human body, but its levels gradually decline with age (Madeo et al., 2018). It is known to combat aging and inflammation primarily by inducing cellular autophagy (Madeo et al., 2018). Spermidine also exhibits therapeutic effects in AD by regulating neuroinflammation, not only at the transcriptional level but also by interfering with the assembly of the NLRP3 inflammasome in microglia (Freitag et al., 2022). Additionally, it can enhance the ability of AD-related microglia to phagocytose Aβ (Freitag et al., 2022). INPP5D is a myeloid-expressed gene that acts as a negative regulator of bone marrow cell proliferation and survival, and it is also a newly identified risk gene for AD (Karch and Goate, 2015; Chou et al., 2023). A decline in the activity of its encoded protein, SHIP1, can induce the formation of the NLRP3 inflammasome in microglia, promoting the secretion of IL-1β and IL-18, thereby inducing inflammation (Chou et al., 2023; Terzioglu and Young-Pearse, 2023). *Polygonum multiflorum* is a traditional Chinese medicinal herb known for its properties in tonifying blood, alleviating dryness, and promoting bowel movements (Liu et al., 2018). One of its active components, tetrahydroxy stilbene glucoside, has been shown to counteract aging and may reduce the activation of the NLRP3 inflammasome in microglia in the context of AD by inhibiting the cyclic guanosine monophosphate-adenosine monophosphate synthase-stimulator of interferon genes (STING) (cGAS-STING) signaling pathway (Gao et al., 2023a). In AD, the NLRP3 inflammasome in microglia is closely related to autophagy. Ubiquitinated NLRP3 can be recognized by the autophagy-related protein p62 and degraded via the autophagy–lysosome pathway, consequently reducing the pro-inflammatory functions of microglia and improving cognitive function (Zhang et al., 2023). Metabolic changes in microglia also play a significant role in AD. When glucose utilization is impaired in the brains of patients with AD, ketone body metabolism is enhanced (Shippy et al., 2025). β-Hydroxybutyrate inhibits the NLRP3 inflammasome in microglia, thereby alleviating neuroinflammation. This suggests that ketogenic therapy may serve as an alternative dietary approach for treating AD (Shippy et al., 2025).

PD is another neurodegenerative disorder characterized primarily by clinical symptoms such as resting tremor, muscular rigidity, postural instability, and bradykinesia (Kalia and Lang, 2015). The pathological hallmark of PD is the degeneration and death of dopaminergic neurons in the substantia nigra. The etiology remains unclear, but is believed to result from a combination of environmental and genetic factors, leading to widespread neuropathological changes and neuroinflammation (Kalia and Lang, 2015). Microglia and inflammasomes play a crucial role in mediating inflammation in PD. NLRP3 is degraded in microglia through chaperone-mediated autophagy (Chen et al., 2021). Impairments in this autophagic process can promote the activation of the NLRP3 inflammasome, aggravating motor dysfunction and neuronal loss in PD (Cheng et al., 2020; Qin et al., 2021b). Parkin, an E3 ubiquitin ligase, is involved in regulating cellular metabolism and mitochondrial autophagy. Some patients with PD exhibit mutations in the parkin gene, which not only disrupt mitochondrial autophagy (Pickrell and Youle, 2015) but also lead to the excessive activation of NLRP3 in microglia, exacerbating the progression of PD (Yan et al., 2023). Andrographolide, as a natural antibiotic, may inhibit NLRP3 activation in microglia by promoting parkin-mediated mitochondrial autophagy (Ahmed et al., 2021). Similarly, urolithin A may also enhance mitochondrial autophagy in microglia, reducing NLRP3 expression and exerting protective effects (Qiu et al., 2022). It is well known that the TREM2 of microglia is significantly associated with AD (Qin et al., 2021a; Wang et al., 2022a). However, research on the relationship between TREM2 and PD is limited. TREM2 reduces NLRP3 activation in microglia through the TLR4/MyD88/NF-κB pathway, exerting anti-inflammatory protective effects (Huang et al., 2024a). Rev-erbα is a circadian rhythm protein and nuclear receptor that regulates the activation of microglia and astrocytes as well as modulates LPS-induced neuroinflammation (Griffin et al., 2019). In PD, the circadian oscillation of Rev-erbα in the substantia nigra is absent, and the activation of Rev-erbα can reduce NLRP3 inflammasome activation in microglia (Kou et al., 2022), which is consistent with neuroinflammation observed in the brains of patients with PD. Notably, defective autophagy in microglia can lead to the overactivation of NLRP3 inflammasomes, which can aggravate motor dysfunction and neuron loss in PD (Choi et al., 2022).

Cerebrovascular diseases are a group of conditions that affect the blood vessels of the brain, resulting in damage to brain tissue due to intracranial blood circulation disorders. These maladies, which are broadly classified and have complex etiologies, lead to significant increase in disability-adjusted life-years each year (GBD 2016 DALYs and HALE Collaborators, 2017). Neuroinflammation has emerged as a crucial factor influencing the prognosis of cerebrovascular diseases, making the study of inflammasomes essential.

Ischemic stroke (IS) refers to the necrosis of brain tissue caused by the narrowing or blockage of arteries supplying blood to the brain, resulting in insufficient cerebral blood flow. This condition imposes a substantial economic and social burden globally (Li et al., 2024b). Specifically, microglia constitute the primary source of inflammasomes following IS, although astrocytes are also involved (Heinisch et al., 2022). After cerebral ischemia, the expression of STING in microglia is increased, promoting the production of NLRP3 (Li et al., 2024a). Suppression of the NLRP3 pathway in microglia mitigates injury resulting from IS in the brain (Du et al., 2020; Ran et al., 2021). Intriguingly, erythropoietin, a drug predominantly employed clinically for treating anemia, alleviates post-ischemic injury such as infarcts and nerve damage in brain tissue by attenuating post-ischemic activation of NLRP3, NLRC4, and AIM-2 inflammasomes in microglia (Heinisch et al., 2022). Besides, erythropoietin ameliorates microglial metabolic abnormalities and prevents cell death after ischemia and hypoxia (Arik et al., 2022). Therefore, the inclusion of anti-inflammatory drugs in the treatment of IS may be considered to enhance patients’ prognosis. N6-methyladenosine (m^6^A) is the most common and extensively studied type of RNA methylation modification, playing an important role in neurological diseases (Chen et al., 2022; Zhang et al., 2022c). Methyltransferase-like 14 is one of the main methyltransferases that catalyze m^6^A modification, and its levels rise in microglia after middle cerebral artery occlusion. This increase promotes the m^6^A modification of *KAT3B* mRNA, which affects STING, leading to enhanced expression of the NLRP3 inflammasome and pyroptosis in microglia (Li et al., 2023c). Trimethylamine N-oxide, a metabolite produced by gut microbiota from dietary nutrients such as choline, can inhibit FTO and IGF2BP2, which in turn suppresses the m^6^A regulatory levels of NLRP3. Ultimately, this promotes activation of the NLRP3 inflammasome in microglia following hypoxia, exacerbating neuroinflammation after IS (Ge et al., 2023). Curcumin is a natural phenolic antioxidant that can inhibit activation of the NF-κB pathway in microglia, thereby suppressing NLRP3 inflammasome activation and reducing the expression of pyroptosis-related proteins, which ameliorates white matter injury following IS (Ran et al., 2021). Ruxolitinib is the first FDA-approved drug for the treatment of myelofibrosis, and it also has potential therapeutic effects for IS. Ruxolitinib helps to reduce neuroinflammation after IS because it inhibits the expression of the NLRP3 inflammasome by suppressing the JAK2/STAT3 pathway in microglia (Zhu et al., 2021). Oxidized mitochondrial DNA can promote NLRP3 activation, while cytidine/uridine monophosphate kinase 2 facilitates the replication of mitochondrial DNA (Xian et al., 2022). Subsequent to IS, the expression of cytidine/uridine monophosphate kinase 2 in microglia increases, thereby promoting neuroinflammation (Guan et al., 2024).

Cerebral venous sinus thrombosis is a specific type of cerebrovascular disease that is more commonly seen in young adults (Capecchi et al., 2018). Following the formation of cerebral venous sinus thrombosis, the cGAS-STING pathway in microglia is activated, leading to an increase in NLRP3 levels and triggering neuroinflammation (Ding et al., 2022).

Subarachnoid hemorrhage (SAH) refers to a clinical syndrome caused by the rupture of pathological blood vessels at the base or surface of the brain, leading to blood directly entering the subarachnoid space. Owing to its chronicity and associated disabilities, SAH often consumes more social resources than IS (Romoli et al., 2023; Lv et al., 2024). Transmembrane G protein-coupled receptor 5 is a membrane receptor primarily expressed in organs such as the intestines, stomach, and liver, which can reduce NLRP3 expression upon activation by bile acids (Guo et al., 2016). Additionally, transmembrane G protein-coupled receptor 5 is also expressed in neuroglial cells such as microglia, where its activation decreases the activation of the NLRP3-ASC inflammasome, thereby improving short-term neurological function (Hu et al., 2021). Sirtuin 1 is a histone deacetylase that plays a role in regulating cellular metabolism and lifespan, while also reducing NLRP3 activation in microglia and promoting their transformation into the anti-inflammatory M2 phenotype, thereby alleviating early brain damage after SAH (Xia et al., 2021). C–X–C chemokine receptor type 4, a member of G protein-coupled receptor superfamily, serves as a chemokine receptor that plays a crucial role in tumor progression (Richardson, 2016) and regulation of cell proliferation and tissue regeneration (Bianchi and Mezzapelle, 2020). Blocking C–X–C chemokine receptor type 4 can inhibit the production of the NLRP3 inflammasome by modulating the activation of NF-κB in microglia (Liu et al., 2023a).

Intracerebral hemorrhage (ICH)—a condition characterized by blood leakage due to the rupture of blood vessels in the brain—is one of the leading causes of death and disability worldwide (Xu et al., 2024). Didymin, a natural flavonoid glycoside, possesses anti-inflammatory and antioxidant effects (Hung et al., 2010; Zhang and RuXian, 2022). Recent studies have shown that didymin can reduce the number of caspase-1- and GSDMD-positive microglia following ICH, inhibit microglial activation, and decrease the expression of the NLRP3 inflammasome, thereby providing neuroprotection (Gu et al., 2022).

Migraine is a highly prevalent neurological disorder that is primarily characterized by throbbing headache with nausea and vomiting. The global incidence of migraine is rising, significantly impacting quality of life (Hovaguimian and Roth, 2022; Fan et al., 2023). The calcitonin gene-related peptide signaling pathway is involved in regulating neuroinflammation in migraine (Nelson-Maney et al., 2024). Migraine has multiple causes; among these, medication overuse induces central hyperalgesia and calcitonin gene-related peptide upregulation through the microglia P2X7 receptor (P2X7R)/NLRP3 pathway (Wang et al., 2023c). Metformin not only suppresses the activation of microglial NLRP3 inflammasomes, but also inhibits the production of calcitonin gene-related peptide (Fan et al., 2024).

Depression is a major public health concern globally, with a prevalence rate of approximately 27.2% that continues to rise annually (Rotenstein et al., 2016). Extensive clinical research highlights the pivotal role of neuroinflammation in depression (Troubat et al., 2021; Guo et al., 2023), with individuals experiencing chronic inflammation being more susceptible to depressive symptoms (Rosenblat et al., 2014; Brites and Fernandes, 2015). Inflammasomes, particularly the NLRP3 inflammasome, are critical mediators in the pathophysiology of depression. Activation of the NLRP3 inflammasome in microglia drives depression-linked neuroinflammation, suggesting that pharmacological interventions targeting microglia may be beneficial (Han et al., 2024). The gut-brain axis is considered a new target for the treatment of various diseases, with gut microbiota capable of modulating depression-associated neuroinflammation in the brain (Carlessi et al., 2021). Betaine is a naturally occurring alkaloid that exhibits antioxidant and anti-inflammatory properties. Supplementation with betaine may exert antidepressant effects by inhibiting NLRP3 inflammasome activation and facilitating the conversion of microglia from the M1 to the M2 phenotype (Zhang et al., 2022b). Docosahexaenoic acid (DHA) and eicosapentaenoic acid (EPA) are two distinct ω-3 fatty acids that play essential roles in the nervous and immune systems (Chen et al., 2024). Both DHA and EPA reduce neuroinflammation and ameliorate depressive-like behaviors by inhibiting NLRP3 inflammasome formation and promoting the polarization of microglia towards the M2 phenotype (Yang et al., 2023). EPA has been shown to be more effective than DHA, with distinct mechanisms of action: EPA prevents inflammasome assembly by inhibiting NLRP3-ASC binding, while DHA reduces the protein levels of ASC and caspase-1, thereby decreasing inflammation (Wang et al., 2022b). Isoliquiritin is an active chemical component contained in the traditional Chinese medicine *Glycyrrhiza uralensis* known for its effects in clearing heat, detoxifying, and alleviating cough and phlegm (Li et al., 2020). Isoliquiritin exerts antidepressant effects by reducing the activation of inflammasome-associated proteins in microglia, such as NLRP3, cleaved-caspase-1, and GSDMD-N, thereby decreasing pyroptosis and protecting neurons (Li et al., 2021c). Mitochondria are associated with the activation of inflammasomes, and the stability of contact sites between mitochondria and the endoplasmic reticulum is crucial for cellular inflammation. Activation of the eATP-P2X7R signaling pathway in microglia disrupts the normal structure and function of these contact sites, facilitating the generation of the NLRP3 inflammasome and contributing to the development of depressive-like behaviors (Zhang et al., 2024a).

Other systemic diseases are closely linked to depression. Studies have shown that a high-glucose environment promotes the assembly and synthesis of the NLRP3 inflammasome in microglia, leading to depressive-like behaviors in diabetic mice (Su et al., 2023). Similarly, mice with allergic rhinitis exhibit depressive-like behaviors, potentially associated with the activation of NLRP3 in microglia within the anterior cingulate cortex (Gao et al., 2023c). Moreover, metformin has been shown to alleviate these depressive-like behaviors, suggesting that, among patients with diabetes, metformin not only lowers blood glucose levels but also reduces neuroinflammation, thereby minimizing the risk of depression-like behaviors (Gao et al., 2023c). Chronic pelvic pain may contribute to the onset of depression by activating microglia in the central nervous system, which promotes the expression of the NLRP3 inflammasome (Mokhtari et al., 2024).

The NLRP1 inflammasome is primarily expressed in neurons, not in microglia, while C–C chemokine receptor 5 (CCR5) activation in microglia induces NLRP1-dependent pyroptosis in neurons (Yan et al., 2021). This underlines the tight association between microglia and neurons, in both physiological and pathological contexts. The AIM-2 inflammasome plays a significant role in microglia. Initially, AIM-2 inflammasomes can generate pro-inflammatory effects. AIM-2 can compensate for NLRP3 abrogation through upregulation of its expression (Heinisch et al., 2022). However, deletion of the *Aim-2* gene has been shown to result in reduced deposition of amyloid peptides and microglial activation, accompanied by increased expression of inflammatory factors, in the brains of a mouse model of AD (Wu et al., 2017). Another study demonstrated that the absence of AIM-2 heightened microglia activation, subsequently promoting neuroinflammation in a mouse model of experimental autoimmune encephalomyelitis (Ma et al., 2021). These results imply that the effects of AIM-2 differ between the central and peripheral regions. This prompts the consideration of distinct pro- or anti-inflammatory “subtypes” in the central region or differing effects in various diseases, necessitating further experiments for verification.

Recently, it has been discovered that noncanonical inflammasomes also play a vital role in microglia. Inhibition of noncanonical inflammasome activation through the TLR4-NF-κB pathway can reduce microglial inflammation and neurotoxic responses (Zhong et al., 2019). In addition, recognition of ligands by TLRs induces activation of the caspase-8 inflammasome in microglia, which in turn contributes to neuroinflammation (Zhang et al., 2018). These findings may indicate a compensatory or complementary relationship between canonical and noncanonical inflammasomes, involving potential positive and negative feedback as well as cascade amplification of signals.

### Astrocytes and inflammasomes

#### Overview of astrocytes and neuroinflammation

Astrocytes, the most numerous types of GCs in the CNS (Freeman, 2010), are characterized by their expression of glial fibrillary acidic protein (GFAP) (Eng et al., 2000; Yang and Wang, 2015). Based on differences in morphology, location, and structure, astrocytes can be divided into four subgroups: fibrous, protoplasmic, interlaminar, and varicose projection, with the first two being the primary subgroups (Miller and Raff, 1984; Kettenmann and Verkhratsky, 2008; Oberheim et al., 2009; Sofroniew and Vinters, 2010; Tabata, 2015; Vasile et al., 2017; Ravi et al., 2021). The normal physiological functions of astrocytes encompass regulation of synapse formation and elimination, promoting neuronal survival, exchanging substances and information with other cells, gliotransmission, participating in the formation of the BBB, and secreting neurotrophic factors (Navarrete et al., 2013; Chung et al., 2015; Zhang et al., 2016; Vasile et al., 2017; Candelario-Jalil et al., 2022; Liu et al., 2022; Sivakumar and Krishnan, 2023). Dysfunction of astrocytes is associated with various CNS diseases, including PD (Kam et al., 2020), Huntington’s diseases (Hsiao et al., 2015) as well as neurodevelopmental disorders (Caldwell et al., 2022).

Previous studies have demonstrated the diversity of astrocyte populations across various regions of the healthy or diseased brain (John Lin et al., 2017; Endo et al., 2022; Sadick et al., 2022). Similar to microglia, reactive astrocytes can be categorized into two types, namely, “A1” and “A2”. A1 exhibits toxic effects, whereas A2 exerts protective effects on the CNS (Liddelow et al., 2017; Fei et al., 2022). Facilitating the conversion of astrocytes from type A1 to A2 is beneficial for recovery following neurological injury and is expected to become a focal therapeutic strategy (Fei et al., 2022). GFAP remains a marker for many, but not all, reactive astrocytes because of variant astrocyte expression influenced by the location and extent of CNS injury (Sofroniew, 2014; Giovannoni and Quintana, 2020). Furthermore, the GFAP level in blood could serve as a marker for various brain and spinal cord diseases, including traumatic brain injury, inflammatory diseases, and neurodegenerative disorders (Abdelhak et al., 2022). This information could be integrated with routine detection methods, such as imaging, in clinical practice to assess patients early, comprehensively, and effectively (Abdelhak et al., 2022). Besides, GFAP is associated with inflammasome activation (Kuo et al., 2022; Lei et al., 2024). Aquaporin-4 (AQP4), a subtype of water-channel protein, is highly expressed in astrocytes and its polarization is implicated in AD (Feng et al., 2023). Interestingly, the number of AQP4 polarizations is correlated with astrocyte phenotype; specifically, neuroprotective astrocytes exhibit a larger number of AQP4 polarizations than neurotoxic astrocytes (Feng et al., 2023). Several studies have demonstrated that AQP4 is closely related to neuroinflammation and inflammasome signaling in various diseases; however, the precise regulatory mechanisms remain unclear (Wang et al., 2020; Xin et al., 2023). AQP4 also mediates brain edema, which may be associated with NLRP3 and other inflammasomes (Lenart et al., 2016; Wang et al., 2020). The development of drugs that target astrocyte markers could potentially reduce inflammasome activation and alleviate neuroinflammation in neurological disorders.

Astrocytes are involved in neuroinflammation through multiple mechanisms (Giovannoni and Quintana, 2020). Dysfunction in the oxidative phosphorylation of astrocyte mitochondria leads to the accumulation of fatty acids, which results in neuroinflammation and AD (Mi et al., 2023). Neuroinflammation and neurodegeneration give rise to various types of reactive astrocytes (Leng et al., 2022; Patani et al., 2023). Two distinct reactive astrocyte populations, responsive to the inflammatory mediators IL-6 and IFN downstream of NF-κB, are both activated in AD. Interestingly, these are opposing responses—IL-6 signaling is promoted by STAT3, while IFN signaling is inhibited by STAT3 (Leng et al., 2022). This suggests that the same downstream signaling pathway leads to two mutually antagonistic responses, both modulated by the same molecule. Altering one of these effects may be a useful strategy in treating diseases.

#### Astrocyte-associated inflammasomes in diseases

Recently, inflammasomes have been found to play essential roles in the astrocyte-mediated inflammatory response in the CNS. Upon stimulation of the brain by pathological factors or in disease states, astrocytic inflammasomes are triggered, leading to the induction of IL-1β and IL-18 secretion, thereby mediating inflammation (Voet et al., 2019). Importantly, elevated IL-1β levels can, conversely, stimulate astrocytes to release additional chemokines, exacerbating neurological damage (Lopez-Rodriguez et al., 2021). Therefore, when caring for patients with CNS inflammation, it is crucial to prevent infection and inflammation in other parts of the body in order to avoid exacerbating the condition.

In addition to microglia, astrocytes also play a vital role in neuroinflammation in the brain of patients with AD. Non-coding RNAs are a class of RNA molecules transcribed from genes that are not translated into proteins, but directly regulate physiological functions. miR-223-3p is considered a negative regulator of NLRP3 (Mancuso et al., 2019), while the long non-coding RNA SNHG14 acts as a sponge for miR-223-3p (Duan et al., 2021). The nonpeptide angiotensin-(1–7) analogue AVE0991 can inhibit NLRP3 expression by reducing SNHG14 expression, thereby increasing miR-223-3p levels and downregulating astrocyte-mediated neuroinflammation, which mitigates neuronal damage and cognitive impairment (Duan et al., 2021). Another microRNA, miR-224-5p, can also suppress NLRP3 expression in astrocytes and its transcription, intriguingly, promoted by diminazene (Sun et al., 2023). P2X7R is associated with the activation of the NLRP3 inflammasome. Interestingly, P2X7R can induce NLRP3 inflammasome production in microglia but not in astrocytes (Beltran-Lobo et al., 2023). A study based on human post-mortem tissue reveals no difference in the levels of NLRP3 produced by activated microglia between control and AD groups. However, the activation levels of astrocytes are significantly elevated in AD (Tang and Harte, 2021). Glucagon-like peptide-1 is a hormone primarily produced by intestinal L cells that effectively lowers blood sugar levels as well as exhibits anti-inflammatory and neuroprotective effects (Li et al., 2021a). The natural analog of the glucagon-like peptide-1 receptor agonist, exendin-4, can reduce the expression of NLRP2 in astrocytes, therefore alleviating neuroinflammation and improving cognitive function (Zhang et al., 2022a). Compared with those of NLRP3, the levels of AIM2, NLRP1, and NLRC4 do not increase in the cortices of AD mouse models (Hou et al., 2021). Recent studies indicate that human cortical astrocytes express the calcium-sensing receptor, which can form complexes with Aβ and activate the TYK2/JAK/STAT signaling pathway, upregulating NLRP2 and NLRP3 inflammasomes and downregulating the AIM-2 inflammasome, leading to neurotoxicity and neuroinflammation. Furthermore, negative allosteric modulators of the calcium-sensing receptor can block the activation of the TYK2/JAK/STAT signaling pathway, providing a new direction for clinical treatment of AD (Chiarini et al., 2025).

The activation status of certain surface receptors on astrocytes also influences the progression of PD. Cannabinoid receptor 2 has been shown to play a significant role in various diseases (Leite-Avalca et al., 2021; Magham et al., 2021; Gao et al., 2023b). Activation of cannabinoid receptor 2 on astrocytes promotes degradation of the NLRP3 inflammasome through autophagic pathways, decreasing NLRP3 levels and alleviating PD symptoms (Zhu et al., 2023). Dopamine receptor agonists are commonly used in clinical settings to treat PD. Pramipexole, one such agonist, can enhance autophagy through the Drd3-related signaling pathway, inhibiting the NLRP3 inflammasome in astrocytes and mitigating neuroinflammation to protect dopamine neurons (Dong et al., 2023). The alanine-serine-cysteine transporter 2 is considered a new target for cancer treatment (Jiang et al., 2020; Saruta et al., 2022) and has been shown to be highly associated with PD. It can bind to NLRP3, promoting astrocytic inflammasome-triggered neuroinflammation. Furthermore, the target drug for alanine-serine-cysteine transporter 2, talniflumate, has the potential to suppress neuroinflammation, offering new hope for the treatment of PD (Liu et al., 2023b).

The inflammasomes in astrocytes also play important roles in cerebrovascular diseases. Lipocalin-2, a secreted glycoprotein, plays a significant role in stroke (Zhao et al., 2023). After IS, lipocalin-2 levels rise; furthermore, its receptor, 24p3R, located on astrocytes, induces an increase in NLRP3 inflammasome levels and pyroptosis in astrocytes, exacerbating cognitive deficits (Li et al., 2023b). Fibrous and protoplasmic astrocytes express inflammasome protein components, inducing inflammation, apoptosis, and pyroptosis under ischemic conditions (Wong et al., 2023). Meanwhile, under hypoxic conditions, the NLRP3 inflammasome causes brain damage by activating astrocytes (She et al., 2022). Compared with microglia, higher levels of NLRC4 are expressed in astrocytes (Freeman et al., 2017). Tumor necrosis factor-stimulated gene-6 is a secreted protein associated with inflammation that exhibits anti-inflammatory and tissue-protective properties. Following SAH, tumor necrosis factor-stimulated gene-6 is primarily expressed in astrocytes, where it protects neurons by inhibiting NLRC4 inflammasome activation and pyroptosis in astrocytes (Ding et al., 2024). In contrast to microglia, NLRP6 inflammasomes are predominantly expressed in astrocytes, but not in microglia, following ICH (Wang et al., 2017). Autophagy can exacerbate inflammation in microglia through TLR4 and worsen secondary brain injury after ICH through NLRP6-mediated inflammation (Fu et al., 2022). These findings suggest that in certain brain diseases, CNS inflammation is primarily mediated by a specific class of glial cells, and is supported by another class of glial cells through a different mechanism. This duality may be a contributing factor to drug resistance or the poor efficacy of some drugs.

Moreover, the activation of the NLRP2 inflammasome in astrocytes during depression suggests the presence of neuroinflammation in certain psychiatric disorders (Zhang et al., 2020). Dehydrocorydaline (13-methylpalmatine), an alkaloid with antitumor and antimalarial properties, can alleviate depressive-like behaviors by inhibiting NLRP3-mediated astrocyte activation (Fang et al., 2022). Potassium channels on astrocytes play a significant role in depression (Cui et al., 2018). Kir4.1 may promote neuroinflammation in depression by positively regulating NLRP3 expression in astrocytes (Song et al., 2021). Conversely, the Kir6.1-containing ATP-sensitive potassium channel negatively regulates NLRP3 expression and pyroptosis in astrocytes (Li et al., 2022a). Intervening in the interaction between potassium channels in astrocytes and inflammasomes holds promise as a new strategy for reducing neuroinflammation and treating depression.

Amyotrophic lateral sclerosis is a neurodegenerative disease characterized by the degeneration of both upper and lower motor neurons, manifesting as muscle weakness, atrophy, and pathological reflexes (Feldman et al., 2022). The NLRP3 inflammasome in astrocytes is activated in amyotrophic lateral sclerosis, and inhibiting NLRP3 signaling may alleviate motor symptoms by decreasing neuroinflammation (Johann et al., 2015). PRKR-like endoplasmic reticulum kinase and inositol-requiring enzyme type 1 of astrocytes can activate lipocalin-2 and NLRP3 inflammasomes to induce morphine tolerance and hyperalgesia (Wang et al., 2024). These findings suggest that the subcellular unit activity and molecules of astrocytes may be related to inflammasome activation. Of note, in triple-negative breast cancer with brain metastases, cells secrete soluble factors to activate NLRP3 inflammasomes and IL-1β in astrocytes, whose mediators promote tumor proliferation (Meszaros et al., 2023). Inhibition of inflammasome activation in astrocytes may be helpful for treating metastatic encephaloma. Different from microglia, AIM-2 inflammasomes have been shown to be activated in astrocytes in experimental autoimmune encephalomyelitis, but IL-1β is not significantly elevated, which seems to play a protective role (Barclay et al., 2022). In schizophrenia, the NLRP3 inflammasomes of astrocytes are dysfunctional, affecting innate and adaptive immunity (Szabo et al., 2025).

## Interactions Between Microglia, Astrocytes and Inflammasome

In certain CNS disorders, intracranial microglia and astrocytes develop interactive relationships that influence disease progression. For instance, the crosstalk between microglia and astrocytes contributes to the clearance of α-synuclein (associated with PD) and Aβ (associated with AD) (Rostami et al., 2021). Microglia express NLRP3 inflammasome-related proteins, including ASC, NLRP3, and caspase-1, while astrocytes do not express ASC or caspase-1 but do express caspase-8. The high expression of NLRP3 in microglia is associated with neuronal loss, exacerbating the progression of AD (Moonen et al., 2023). When AD mice have secondary inflammation or infection, activated microglia are recruited around the Aβ plaque, NLRP3 inflammasomes are assembled, IL-1β is activated, and, subsequently, astrocytes express higher levels of chemokines and exhibit a stronger inflammatory responses (Lopez-Rodriguez et al., 2021), which demonstrates that microglia and astrocytes establish connections through inflammasome. In some cases, connections between microglia and astrocytes may produce more neuroinflammation and worsen the condition. Another study showed that cleaved GSDMD is present in both microglia and astrocytes in AD. However, all components of the NLRP3 inflammasome can only be detected in microglia, and astrocytes expresses caspase-8. Notably, microglia and astrocytes that exhibit cleaved GSDMD are located near Aβ plaque in AD (Moonen et al., 2023), which further indicates a close relationship between inflammasome-associated microglia and astrocytes, with an important role in the development of this disease. Additionally, NAD^+^ is an essential coenzyme involved in metabolism, regulation of cellular functions, and anti-aging processes. In AD, NAD^+^ levels are significantly decreased, and its associated metabolic pathways are severely impaired (Lautrup et al., 2019). One of the precursors of NAD^+^, nicotinamide riboside, has been shown to inhibit the activation of microglia and astrocytes, reduce NLRP3 expression, and alleviate cellular damage and aging, making it a potential drug for slowing the progression of AD (Hou et al., 2021). *Porphyromonas gingivalis* is a gram-negative anaerobic bacterium and key periodontal pathogen. Some studies have suggested its association with the pathogenesis of AD (Dominy et al., 2019; Costa et al., 2021), although the specific mechanisms of action remain unclear. The outer membrane vesicles of *Porphyromonas gingivalis* can activate microglia and astrocytes in the brain, as well as trigger the activation of the NLRP3 inflammasome within microglia, promoting the progression of inflammation and leading to memory impairment (Gong et al., 2022). These findings suggest novel therapeutic targets for AD.

Serum and glucocorticoid-regulated kinase 1, a member of the serine/threonine protein kinase family, is elevated in various neurodegenerative diseases. Serum and glucocorticoid-regulated kinase 1 can exacerbate neuroinflammation by activating the NF-κB pathway in microglia and astrocytes, and enhance NLRP3 inflammasome expression, making it a potential therapeutic target in PD (Kwon et al., 2021). Interestingly, the activation of microglia and astrocytes exacerbates the progression of neuroinflammation in the early phase of IS, but and inhibits the spread of inflammation as a result of the formation of glial scars in the late phase (Liang et al., 2023). Following IS, AIM-2 inflammasome activation is involved in mediating post-stroke cognitive impairment. Interestingly, it is primarily expressed in microglia rather than astrocytes (Kim et al., 2020). The expression levels of NLRP1, NLRC4, and AIM-2 increase in astrocytes after hypoxia, which may indicate a time-dependent expression of inflammasomes in different cell types. In ICH, the expression levels of the NLRP3 inflammasome increase in both microglia and astrocytes (Xiao et al., 2023). CCR5 is another type of chemokine receptor that plays significant roles in diseases such as HIV infection (Lederman et al., 2006), COVID-19, and cancer (Zeng et al., 2022). Following brain hemorrhage, CCR5 expression rises in both microglia and astrocytes, along with an increase in CCL5 levels. The activation of CCR5 promotes NLRP1-mediated pyroptosis through the PKA-CREB pathway (Yan et al., 2021). The NLRP3 inflammasome in microglia promotes the activation of caspase-1, which, in turn, induces the transformation of astrocytes into A1 neurotoxic reactive astrocytes, leading to neurological dysfunction and dendritic damage. Interestingly, NLRP3 in astrocytes does not appear to influence the induction of A1-like astrocytes, suggesting that glial cells can interact with each other through inflammasomes (Li et al., 2022c). Fecal microbiota transplantation has been shown to alleviate depressive-like behaviors by inhibiting the activation of microglia and astrocytes, thereby reducing the expression of the NLRP3 inflammasome (Rao et al., 2021). Similarly, in endotoxemia, the NLRP3 inflammasome of microglia facilitates astrocyte conversion to type A1, damages neurons, and reduces cognitive function (Xiao et al., 2022).

The potential connection between microglia and astrocytes through inflammasomes in other neurological diseases requires further study. Additionally, subcellular structures may have intricate relationships with inflammasomes, warranting further investigation.

## Limitations

This review has some limitations. Our scope is limited as we have not explored the relationship between inflammasomes and other CNS glial cells, such as oligodendrocytes, and we have not addressed peripheral nervous system glial cells. Furthermore, inflammasomes in glial cells may impact not only neurons but also vascular endothelial cells, potentially influencing clinical signs and symptoms following cerebrovascular accidents. Besides, it is yet to be investigated whether inflammasomes in the nervous system contribute to systemic inflammation.

## Conclusions

In this review, we delineate the classification and activation of inflammasomes; further, we describe the mechanisms by which canonical and noncanonical inflammasomes are regulated, with a focus on two crucial glial cells in the CNS—microglia and astrocytes. We explore the association of these two cell types with inflammasomes. Inflammasomes are divided into canonical inflammasomes and noncanonical inflammasomes. Canonical inflammasomes include NLRP3, NLRP1, NLRC4, NLRP6, and AIM-2, which can induce neuroinflammation by activating caspase-1 and, subsequently, IL-1β and IL-18, while noncanonical inflammasomes activate caspase-11.

Among the glial cells, microglia and astrocytes are most closely associated with neuroinflammation in the CNS and play crucial roles in various neurological disorders, including AD, PD, and cerebrovascular diseases. Neuroinflammation, as a double-edged sword, is widely present in these diseases. Neuroinflammation activates microglia and astrocytes to promote the phagocytosis of harmful substances; on the other hand, it leads to the continuous release of inflammatory factors that exacerbate brain damage (Kennedy, 2015). Inflammasomes, as key regulators of neuroinflammation, play an important role in the CNS. By modulating the inflammasomes in microglia and astrocytes at different stages of diseases, it may be possible to delay disease progression, improve prognosis, and promote recovery, offering a potential new direction for clinical therapy. The NLRP3 inflammasome may be the most extensively studied and well-understood inflammasome type in terms of its mechanisms (Paik et al., 2021; Fu and Wu, 2023). However, the influence of other inflammasomes on the CNS should not be overlooked, and our understanding of their precise mechanisms and therapeutic potential remains limited. This review highlights the effects of various substances on the activation or inhibition of inflammasomes in microglia and astrocytes, although the detailed mechanisms remain unclear. Future research should focus on elucidating the precise molecular mechanisms underlying inflammasome regulation and their roles in disease progression, with the aim of developing targeted drugs to treat diseases while minimizing side effects. Additionally, the potential roles of inflammasomes in the BBB and brain edema represent a promising research area. Furthermore, future studies should focus on understanding how the crosstalk between microglia, astrocytes, and inflammasomes influences inflammasome activation and how these interactions can be leveraged for therapeutic benefit. Selectively targeting inflammasomes in microglia or astrocytes without disrupting their essential roles in maintaining CNS homeostasis remains a significant challenge for future research. Finally, the potential therapeutic utility of modulating microglial and astrocytic inflammasome activity through pharmacological agents, regulation of autophagy, or immune modulation, particularly in neurodegenerative and neuroinflammatory diseases, should be further explored.

We expect that this review will contribute a deeper understanding of the pathogenesis of neurological disorders and inflammatory diseases, offering novel ideas and approaches for clinical treatment and drug utilization. The roles of microglia and astrocytes in neuroinflammation have been extensively documented. Future research should prioritize more in-depth mechanistic and pharmacological studies to further elucidate the links between neurons in disease.

## Data Availability

*Not applicable*.
